# Transitioning from face-to-face treatment to iCBT for youths in primary care – therapists' attitudes and experiences

**DOI:** 10.1016/j.invent.2020.100356

**Published:** 2020-12-01

**Authors:** Sandra Weineland, Rasmus Ribbegårdh, Marie Kivi, Andreas Bygdell, Anna Larsson, Kristofer Vernmark, Josefine L. Lilja

**Affiliations:** a Department of Psychology, University of Gothenburg, Gothenburg, Sweden; b R&D Primary Health Care, Västra Götaland, Sweden; c Department of Behavioral Sciences and Learning (IBL), Linköping University, Linköping, Sweden

**Keywords:** iCBT, Adolescents, Children, Implementation, Therapists, Qualitative, Experiences

## Abstract

**Background:**

To address the increasing mental health problems among young people, health care needs to broaden the spectrum of interventions and increase access to care. One particularly promising first-line intervention is cognitive behavioral therapy (CBT) delivered via the Internet (iCBT). The outbreak of the Coronavirus disease -2019 (COVID -19) has made the need for solid digital mental health care systems clear. This is the first published study exploring the transition among therapists of working with face-to-face treatment to using iCBT for youths suffering from anxiety treated in primary care.

**Methods:**

Fourteen primary care therapists were included in the study. Semi-structured interviews (n = 26) were conducted on two occasions: before starting to use iCBT for youths, and at a subsequent follow-up after gaining treatment experience. Data was summarized into thematic categories.

**Results:**

The overarching themes that were identified were: Attitudes to iCBT before and after implementation; Experiences of treatment delivery; Characteristics of “the right patient;” and The role of the digital therapist.

**Conclusion:**

The participants generally had positive attitudes to iCBT for youths and saw it as a valuable alternative to face-to-face treatments. However, they identified challenges related to patient selection, and to motivating patients and maintaining a therapeutic relationship through mainly written communication. The participants appreciated the increase in variety that iCBT brought to their schedules, and also experienced iCBT as a relief from common challenges of therapeutic work, such as emotional stress and high cognitive demands. The participating therapists' positive experiences support the introduction of iCBT for youths in routine primary care.

## Highlights

• Therapists see iCBT as a valuable addition among treatment methods in primary care.
• According to therapists, a successful implementation of iCBT depends on a clear rationale for iCBT.
• Therapists described positive effects of high parental involvement in iCBT for adolescents.
• In the transition to iCBT, therapists experienced a relief from common challenges of therapeutic work face-to-face.

## 1 Background

Over the last decade, mental health problems among Swedish children and young people have doubled (National Board of Health and Welfare, 2017), with comparable increases reported in other countries, such as the USA (Twenge et al., 2019). Anxiety is the most common form of mental health problems in minors (Thapar et al., 2017). Children and adolescents should receive treatment early; otherwise their problems are at risk of becoming chronic (Sawyer et al., 2012).

Cognitive behavioral therapy (CBT) is a well-documented and effective method for anxiety and depression in adolescents, and is considered the treatment of choice by the Swedish National Board of Health and Welfare (2017). However, both for adults and children, access to CBT is limited, due to factors such as high cost of treatment, long waiting lists, and lack of therapists in rural areas (Jolstedt et al., 2018). In addition, many adolescents avoid seeking care for mental health issues due to a lack of knowledge, perceived stigma, long waiting times, or geographical distances (O'Dea et al., 2015).

To cope with increasing mental health problems in children and adolescents, the range of interventions in primary care needs to be expanded. One way to increase access the range of interventions in primary care is providing stepwise care, meaning that a patient receives the least resource-demanding evidence-based treatment that is appropriate for his or her diagnosis and symptom severity. After assessment, patients with lower levels of functioning can directly move on to more intensive, individualized treatments, while patients with a higher level of functioning can be offered a first-line, low-cost intervention, thus freeing up resources for more complex cases (Yan et al., 2019). One particularly promising first-line intervention is CBT delivered via the Internet (iCBT). Cognitive behavioral therapy delivered via the Internet shows treatment effectiveness comparable to that of CBT (Andrews et al., 2018; Carlbring et al., 2018). The treatment is also considered to be cost-effective (Ophuis et al., 2017).

There is a need for solid mental health care systems in times of public health emergencies such as the outbreak of the Coronavirus disease -2019 (COVID -19) (Wind et al., 2020). To reduce the risk of spreading infections many health care practitioners' turn to digital therapies. There seem to be a research gap in the research knowledge of the transition of face-to-face therapies to digital treatments among mental health care clinicians working in primary care with youths. The current study is the first published study that explore this transition.

Successful implementation of iCBT has been studied in a large-scale study of five specialized iCBT clinics in Scandinavia and Australia (Titov et al., 2018). The authors considered a couple of factors that contribute to successful implementation of iCBT in regular care, namely, that: management and organization should prioritize implementation; specialist trained practitioners and evidence-based and validated digital treatment programs must be available; ongoing evaluation of patient satisfaction and treatment outcomes must be supported; and there must also be easy paths into care (e.g., self-referrals) as well as clear routines for triage and assessment (Titov et al., 2018).

To date there is an ongoing wide-spread success of iCBT in anxious youth populations. Studies have shown that iCBT can reduce the perceived stigma about seeking psychological treatment among adolescents and, thus, further increase access to effective care (O'Dea et al., 2015). A recent published study in the Swedish context showed reduced anxiety and depressive symptoms among adolescents receiving iCBT (Berg et al., 2020). Two meta-analyses have indicated that iCBT for child and adolescent anxiety is effective (Ebert et al., 2015; Vigerland et al., 2016). However, iCBT for youths is still considered to be in a developmental phase regarding implementation (Jolstedt et al., 2018).

The attitude to iCBT among practicing therapists is an important factor in successful implementation of iCBT for youths in primary care. Primary care practitioners are largely positive to iCBT, but many perceive it as less effective than conventional treatment (Perle et al., 2013). In a survey of Swedish practitioners' attitudes to iCBT for youths, Vigerland et al. (2014) found that therapists consider iCBT for young people to be as effective as face-to-face treatment of mild to moderate problems and believe that it can contribute to more young people receiving care. Results also showed that therapists are concerned about a lack of alliance in iCBT, and that iCBT should not be given to patients with overly complex problems. The vast majority (86%) in this study had no experience of working with iCBT (Vigerland et al., 2014). To expand on previous research and explore the specific transition of providing face-to-face care to iCBT with youth anxiety, practitioners' subsequent experiences pre- and post-implementation of iCBT might be important to explore. Qualitative insights might be needed to better understand the process of transitioning from face-to-face to internet delivered treatment (Folker et al., 2018). The present study specifically aims to explore the transition from working with face-to-face treatment to using iCBT for youths suffering from anxiety treated in primary care. Therapists' attitudes were examined on two occasions: before starting to use iCBT, and at a subsequent follow-up after gaining treatment experience. The research questions were the following:

1. What are the attitudes and experiences to iCBT among practicing therapists before and after implementation of iCBT for youths in primary care?
2. What are the challenges to consider while implementing iCBT for youths in primary care?
3. What are the experienced changes in the transition from face-to-face therapy to iCBT for youths in primary care?
4. What are the main factors to consider when implementing iCBT for youths in primary health care?

## 2 Methods

The implementation of iCBT was part of a regional implementation and research project conducted during 2017–2020 (Swedish National Research Register (FoU), ID No.: **240221**). The study was approved by the Regional Ethics Committee in Gothenburg (Dnr: 703–17). The patients, adolescents and parents treated by the participating therapists of the current study, sought help for mild to moderate anxiety within primary care. A guardian was present at assessment and at follow-up talks. All patient was assessed by means of a clinical interview and semistructured interview MINI-KID and the child and guardians completed the pre-measurements. All patients were provided written parental and patient consent and verbal assent since they participated in the larger project, mentioned above, implementation of iCBT conducted during 2017–2020. The treatment period were 8 weeks. During the return visit, the participant and parent met the treatment therapist for a final interview and filling-in self-assessment scales digitally.

### 2.1 Study design

A qualitative study using semi-structured interviews with 14 therapists from eight mental health centers was conducted between January 2017 and December 2019. Some of the health centers are located in rural areas and the others in the city of Gothenburg.

### 2.2 Recruitment and study population

Therapists were invited through eight Youth Mental Health Centers (in Swedish: *Ungas Psykiska Hälsa*) in primary care, in Västra Götaland region, Sweden, to attend a 2-day course in iCBT for youths. The inclusion criteria were: licensed psychologists, psychotherapists, social workers, or graduated psychologists under supervision before being licensed, with training and documented CBT skills, as well as training in the national Support and Treatment Platform (STP); and attending a 2-day course in the iCBT treatment program *Anxiety Help for Adolescents* (in Swedish: *Ångesthjälpen Ung*). Oral and written information about the study was given on day 1 of the 2-day course. Participants accepted participation in the study by signing the consent form.

The study participants were 14 therapists in primary care in Västra Götaland in Sweden, who worked with psychological treatment of mental health problems in children and adolescents. Thirteen participants were psychologists, three of whom were senior specialist psychologists and two of whom were graduated psychologists under supervision before becoming licensed psychologists. One participant was a social worker with basic psychotherapy training in CBT. Seven of the participants stated that they had been trained in CBT; six were trained in both CBT and psychodynamic therapy, and one only in psychodynamic therapy. The majority, eight participants, were relatively new to the profession (0–5 years of experience), while two had longer experience, of 16–20 years or more. The participants' median age was 36–45 years (six participants were 23–35 years of age; six were 36–45; and two were 46–55 years old). Eleven of the participants were women and three were men. Three participants had previously worked with iCBT for adults. The other eleven participants had no previous experience of Internet-delivered psychological treatment.

### 2.3 Intervention

All participating therapists received training in the iCBT program for youths, which is a guided Internet-delivered self-help treatment program developed by Psykologpartners W&W AB (Linköping, Sweden). *Anxiety Help for Adolescents* is a transdiagnostic program aimed at youths between the ages of 13 and 19, suffering from mild to moderate anxiety. The intended treatment period is 8–10 weeks including assessment and evaluation. The program relies heavily on exposure therapy as well as Acceptance and Commitment Therapy (ACT) as described in a treatment manual developed by Hayes et al. (2011). Interventions based on ACT include mindfulness, acceptance of difficult feelings, and valued action. The content has been adapted to match the particular needs of adolescents according to the model developed by Hayes and Ciarrochi (2015). Patients are asked to complete one module per week in a predetermined order. The patients engage in weekly written communication with their therapist, who provides feedback on treatment progress, answers questions, and offers encouragement. Treatment effects were measured with the Revised Children's Anxiety and Depression Scale (Chorpita et al., 2015) at pre-, mid- and post-treatment. Also, patients answered four questions regarding anxiety, valued action, life quality and homework assignments each week during the course of treatment, which the therapist used for evaluation of progress.

### 2.4 Procedure

Data were obtained through two semi-structured interviews conducted by phone. Interviews lasted approximately 30–60 min per interview. The first of the two interviews was conducted before the therapists started using the iCBT programme, hereafter referred to as “pre-interview.” The second interview was conducted after the participants had treated a minimum of one to two patients, and is hereafter called “post-interview.” The interviews were conducted by the authors, four clinical researchers and research assistants in the study (A.B., A.L., J.L.L., and S.W.). Interviews were based on an interview guide containing open questions such as “What advantages and disadvantages have you found with iCBT compared to other forms of treatment?,” “What treatment results have you been able to observe?,” and “Have you identified any obstacles, either for you or for your patients?” The interviews were audiotaped and transcribed verbatim.

### 2.5 Data analysis

A qualitative content analysis was conducted on 26 transcribed interviews (14 pre-interviews and twelve post-interviews). The material was read several times, and was then descriptively coded based on its manifest meaningful content in accordance with thematic analysis (Willig, 2013). This means that pieces of the raw data, the interview material, were linked to descriptive labels, so-called “code names.”

The analysis was performed by more than one of the researchers to ensure reliability and trustworthiness. Two of the authors (A.B. and R.R.) started to analyze the data and then had regular meetings with the co-authors and supervisors M.K., J.L.L., and S.W. Materials that did not yield expressions of attitudes were not coded. Text that expressed attitudes to, or experiences of, several aspects of iCBT were coded with two or more code names. The codes were hierarchically grouped into larger themes. The themes selected and presented were considered to best illustrate the attitudes or experiences expressed by the participants. Methods of resolving disagreement between authors were to discuss the themes in depth until agreement was reached. Thus, the qualitative analysis was conducted by the team of authors. All authors' perspectives are those of clinical psychologists with knowledge of both the cognitive behavioral approach and iCBT. The clinical expertise of all the authors and researchers enriched the study's data interpretation through in-depth discussions on the possible meanings of text in the context of implementation of iCBT in primary care.

## 3 Results

Based on the qualitative results, four main themes were generated, with associated subthemes (see Table 1). The quotes in the text have been translated into English by the authors.

**Table 1:** Attitudes to, and experiences of, implementation of Internet-delivered cognitive behavioral therapy (iCBT) for adolescents in primary care – themes and subthemes.

Themes	Subthemes
1. Attitudes to iCBT before and after implementation	1.1 Confidence in iCBT vs. doubt regarding organizational motivation
	1.2 Quality assurance and equal care
	1.3 Accessible and non-stigmatizing
2. Experiences of treatment delivery	2.1 Increased efficiency with maintained quality
	2.2 Satisfactory treatment results
3. Characteristics of “the right patient”	3.1 Assessment is paramount
	3.2 Parental support could compensate for low motivation
4. The role of the digital therapist	4.1 More variety, less emotional and intellectual strain
	4.2 The therapist remains important in digital treatments
	4.3 Written communication entails new therapeutic challenges

### 3.1 Attitudes to iCBT before and after implementation

#### 3.1.1 Confidence in iCBT vs. doubt regarding organizational motivation

Most participants expressed positive attitudes towards iCBT and its implementation in primary care. Some, however, believed that colleagues who were skeptical of iCBT may have chosen to remain quiet during the interview. Others expressed that they, or their colleagues, had been skeptical in the past but were more positive after receiving training. The participants collectively expressed confidence that iCBT can have good treatment results.

The treatment program in the study, *Anxiety Help for Adolescents*, was described by the participants as modern CBT with certain third-wave CBT and ACT elements. The participants perceived the program as well designed, educational, and adapted for the target group. Although, in some instances, the treatment program uses its own terminology, the participants agreed that the contents do not differ from conventional ACT-inspired CBT treatments.

Parallel to the positive attitudes, some more cautious attitudes were expressed. None of the participants said that they are explicitly opposed to the introduction of iCBT as such, but some expressed concerns about organizational motivations for introducing iCBT. There were fears that iCBT is being implemented in health care primarily to meet the needs of the organization for a quick, cheap, and standardized intervention.

It would be unfortunate if because of efficiency concerns you try to push as many people into it as possible, to make it happen as quickly as possible, and then many people feel that it was not right for them and it's not going well for them. – Participant 2, pre-interview.

Prior to implementation, some participants expressed a lack of trust in information technology (IT), based on the concern that it could at any time interfere with their work. In the second round of interviews, however, it became clear that technical issues did not constitute a problem. Even therapists with limited IT experience expressed satisfaction with the treatment platform's stability and ease of use.

#### 3.1.2 Quality assurance and equal care

The participants described iCBT as a structured treatment with a predetermined course. Some participants considered the structured treatment format as a consequence of the digital medium and compared it to a strictly manualized CBT treatment.

The division into modules and the standardization of content was predominantly seen as positive. The clear structure of the program was perceived to ensure that all important treatment components are delivered, and that the patient receives evidence-based and equivalent care.

When you create a program this way, the level of quality is secured. Patients receive equal care that is based on a model that they believe works. In this way, treatment quality is always assured, which is not the case with face-to-face treatments. – Participant 3, pre-interview.

The structure of the treatment program was also considered to have a pedagogical function for the patients, as it enables them to easily see what they have been working on and what progress they have made.

Nevertheless, the standardized structure was seen by some participants as somewhat problematic. Their objection was that in this type of treatment you cannot follow a more dynamic course although this, according to the participants, is beneficial, even necessary, when treating certain patients. Most participants, however, saw both a need and a possibility to somewhat tailor all treatments including iCBT to better fit the individual patient. One reason for this was the notion among participants that although many patients appreciate the clear structure, some patients experience the standardization of the program as reductionist. There was significant disagreement as to how much individualization of iCBT treatments there should be. Some wanted to adhere strictly to the treatment program structure, while others were prepared to make major individual adjustments.

#### 3.1.3 Accessible and non-stigmatizing

Some participants believed that iCBT is suitable for patients who have difficulty visiting a clinic, and that this might render therapy accessible to those who would otherwise go without treatment. Such patients' barriers to care can be concrete, such as the travel to the Youth Health Care Center or time-pressured school schedules. Patients' hurdles to conventional face-to-face treatment can also be psychological in nature, as seen, for example, in cases of social phobia. In such instances, iCBT may be a more acceptable treatment since it requires less exposure to anxiety-provoking situations, at least initially. In a similar manner, some participants also believed that some young people may feel intimidated or even stigmatized when consulting a psychologist.

I think iCBT is potentially less stigmatizing than to come and talk to someone. […] When it is easily accessible and without closed doors, I think it is not so shameful. You are being able to sit wherever you want and work on this, you don't have to feel that it is something strange and stigmatizing. There is nothing you need to hide. – Participant 3, pre-interview.

Some participants had perceived a trend in society where mental health is more openly spoken about. Internet-delivered CBT is considered to fit into this trend, as it takes therapy out of the psychotherapist's consulting room into young people's everyday lives. In *Anxiety Help for Adolescents*, exemplifying various anxiety problems through the use of fictional but realistic characters is perceived to contribute to this normalization and can create a sense of group belonging.

### 3.2 Experiences of treatment delivery

#### 3.2.1 Increased efficiency with maintained quality

Most participants considered iCBT to be time efficient. They said that, in order to attain maximum time efficiency, however, therapists and organizations need to learn new ways of working. Most participants expressed that one particular benefit of iCBT is being able to treat a greater number of patients. The weekly amount of time spent on each patient, compared to face-to-face CBT, is significantly reduced, while quality of treatment is maintained, at least when working with patients well suited to iCBT. The therapists felt that the more they used the program, the less time-consuming it became. However, in cases where a therapist treated only a small number of patients in iCBT, the treatment was not believed to contribute to time efficiency. In sum, most participants believed that iCBT allows more patients to receive care with fewer delays, and iCBT was therefore considered a time efficiency treatment modality.

Many participants believed that, for patients suited to the treatment, iCBT can be more efficient than standard CBT, as the treatment platform is available at any time, and accessible from any place. There was a shared opinion among all participants that, when working with patients for whom iCBT works less well, time efficiency drops, as the therapist is required to complement the treatment with repeated notifications, phone calls, and physical appointments meant to motivate the patient and individually tailor the treatment to the non-responding patient. The participants reported that, because of poor patient involvement, iCBT treatments are sometimes prolonged, taking up to twice as long as intended to complete.

#### 3.2.2 Satisfactory treatment results

Most of the interviewed therapists were satisfied with the treatment results in patients who completed the program. The program seems to have enhanced the patients' understanding of themselves and their problems. The participants further reported that the patients had obtained useful strategies to better cope with their symptoms of anxiety. Further reports of improvements include a more active lifestyle, fewer absences from school, and stronger interpersonal skills. Some participants also reported improvements in family relationships.

Of the many skills and strategies taught, many patients had reportedly taken one or two to heart, focusing on them and sometimes failing to implement the others. Most patients had understood and successfully used standard CBT techniques. Likewise, the participants reported that many patients made constructive use of the method of exposure with response prevention (ERP). Regarding interventions that draw more heavily on ACT, rather than standard CBT, the therapists reported mixed results. Certain central ACT concepts, like that of valued action, were perceived as readily accepted and utilized among the adolescent patients. Some patients also seemed to grasp, and benefit from learning, ACT concepts such as mindfulness, acceptance, and cognitive defusion, reporting a certain mindful detachment from their symptoms. At the same time, some participants expressed uncertainty as to whether these mindfulness techniques are accessible enough, and even questioned whether these concepts are suitable for adolescents.

Letting go, being OK with the suffering, the pain: I feel in a way this is something people need to grapple with for years to accomplish […] it's not an easy task, well, intellectually it's not very hard, but truly doing it, emotionally, I'm not too sure that's very easy for adolescents, or that they're even particularly open to such ideas. – Participant 6, post-interview.

Some participants expressed concern that their patients' problems were partly due to circumstances and relational patterns in the family environment and that the clear individual focus on the child in iCBT may make it difficult to identify and work with such family factors.

### 3.3 Characteristics of “the right patient”

#### 3.3.1 Assessment is paramount

All participants discussed what patients can be helped by iCBT. Both before and after the implementation of iCBT, the participants underlined the importance of careful patient selection. The term “the right patient” was used repeatedly to describe which patient is suited and which is not suited for treatment. All participants shared experiences of difficulties, and even failure, in treating patients for whom iCBT was less suitable than traditional face-to-face CBT.

There was considerable consensus among the participants as to what facilitates good treatment results, and as to what might cause obstacles in the treatment process. The participants reported better outcomes with patients who can maintain consistent discipline, accept personal responsibility, and enjoy working in an independent manner. It is also, according to the participants, important that the patient suffers primarily from anxiety, rather than depression, and that his or her symptoms are not too severe. Although the treatment program is designed to be transdiagnostic, the participants believed that patients with more wide-ranging and long-standing problems should probably be excluded.

Many participants also pointed out that the treatment relies on introspection and reflection, which assumes that the patient is capable of, and interested in, using his or her own cognitive resources.

If you're more of a thinker and a bit more goal-oriented, and you need to work through your difficulties in a cognitive manner, then it may work well. – Participant 9, post-interview.

Several participants expressed that, because of the cognitive emphasis, patients with learning difficulties, neuropsychiatric conditions, and/or dyslexia might find the program difficult to follow. Participants further felt, based on developmental psychological theories on child development, that patients should ideally be in their upper teens.

Some participants pointed out that conventional CBT might be a better choice for specific groups of patients. Such groups included patients in need of focused behavioral interventions, such as exposure therapy face to face with a therapist. Some participants also said that, for patients with clearly delineated problems of limited severity, the iCBT program is sometimes unnecessarily long and comprehensive, and that a conventional treatment for those patients might be completed in just a few sessions, compared to the 8 weeks needed for *Anxiety Help for Adolescents.*

#### 3.3.2 Parental support could compensate for low motivation

The participants agreed that iCBT for adolescents puts a lot of responsibility on the patient, which was seen as potentially problematic. The participants felt that iCBT allowed patients with low motivation, patterns of avoidance, and/or procrastination tendencies to postpone or skip treatment activities more easily. However, the heavy responsibility put on the patient was not only seen as negative. Several participants felt that patients who do assume responsibility will experience a clear feeling of mastery when they see that they have done most of the work themselves. Nevertheless, the participants believed that for most patients, assuming full responsibility for their treatment is not possible.

Most participants therefore underlined the importance of consistent parental support for compliance and good treatment results, as well as a stable and secure home environment. Parental support was also perceived as very important as it was believed to greatly decrease the risk of dropout. Involving parents was further perceived as especially crucial for younger patients (13–14 years of age), since their limited knowledge and cognitive capabilities were thought to make the treatment program harder to understand and follow independently.

I have several patients whose parents will sit down with them [at the computer]. If the patient is a bit too young, parents can provide support. The program is intended for 13 years upwards and I wouldn't recommend it to a regular 13-year-old, [or] in that case only with a parent. It requires certain cognitive capabilities that I doubt most 13-year-olds possess. […] Sometimes, if there is uncertainty as to whether the teenager is capable of learning this way, a parent can join in … – Participant 10, post-interview.

Nevertheless, despite the perceived advantages of parental involvement, many patients reportedly preferred their parents not to be involved. Also, not all patients' parents were willing to participate. The lack of clear guidelines on parental involvement was a source of collective concern among the participants. For these reasons, some participants actively tried to find ways to motivate both patients and their parents to take an active, cooperative stance. In sum, all participants saw parental support as valuable, and many expressed the wish for a supplementary treatment component directed at parents. In several cases, success in treatment was attributed specifically to patients and parents working together on the program.

I've noticed that parental involvement actually means quite a lot. Those of my patients who don't allow their parents to participate, or whose parents don't show any interest, those patients don't make as much progress. Having supportive parents is a crucial factor. – Participant 7, post-interview.

### 3.4 The role of the digital therapist

#### 3.4.1 More variety, less emotional and intellectual strain

Before starting to use iCBT, all participants expected that their role and conditions as therapists would change with therapy delivered via the Internet. Many participants expressed a hope that iCBT would enable them to work more flexibly, that is, remotely and according to their own schedule. There were also expectations that iCBT would increase task variety. Some participants expressed hopes that iCBT would lessen their emotional strain, since it was seen as less emotionally demanding than face-to-face treatments. However, there were also concerns that therapists would miss out some of the emotionally most rewarding part of their profession, for instance sharing the feelings of happiness and accomplishment with patients when they improved in treatment.

It becomes less of a personal contact, I think. It's a bit sad, because you won't get the same reward as you do when you meet a patient who is doing better. On the other hand, you do not become as gloomy, perhaps, by chatting with someone who feels bad, as you do when you meet them. – Participant 2, pre-interview.

The post-implementation interviews corresponded well to the participants' predictions. In general, the participants felt that iCBT improved their work by increasing task variety and lessening the stress sometimes associated with face-to-face psychotherapy. Using a treatment program with a pre-determined structure was seen as a cognitive relief, as the time and energy normally spent on case conceptualization and session preparation could be cut drastically. Some participants stated that their usual treatments were improved and facilitated by the information and concepts they had learned through the program.

The flexibility of iCBT was seen as both potentially valuable and problematic. Since iCBT work can easily be rescheduled or postponed, there was a perceived risk that iCBT will be downgraded and squeezed into gaps between more pressing responsibilities. Clearly, careful scheduling of iCBT work becomes a strategy for dealing with both problems, both for their own part and towards management and the organization.

#### 3.4.2 The therapist remains important in digital treatments

Before implementation, some participants expressed that in iCBT, the therapist becomes less important, and replaceable. On the other hand, several participants said that they did not believe that fully automated treatments can be effective, and that the therapeutic relationship will remain important. Post-implementation, the participants agreed that in iCBT, most patients need a therapist present who follows up on progress, provides validation, and offers encouragement. In fact, the participants felt that the therapist needs to be more motivational and supportive than in face-to-face treatments. Many participants further reported that they often needed to assume the role of intermediary between the treatment platform and the patient. A common experience was that the patient did not fully understand the contents of the program, or that it needed to be tailored, even expanded, to match his or her specific needs and make concrete therapeutic change possible.

It's about turning the program into an individualized treatment. They sometimes don't understand just from following the program. The therapist needs to intervene and make personalized comments to the participant in order to clarify. Then they understand, but often the program doesn't really work on its own. – Participant 7, post-interview.

Some participants expressed an expectation, pre-interview, that motivation would be even more crucial in iCBT than in conventional CBT, and that the therapist would play a key role in motivating the iCBT patient. This proved to be true, with many reports, post-interview, of deviations from the standard treatment arrangement (1 module per week). The general impression among the participants was that compliance was lower in iCBT than in standard, face-to-face CBT. There was no consensus as to how to handle patient inactivity, although many participants described ambitious attempts to increase patients' motivation. Other participants took a more passive stance, as they felt that adding telephone calls and even clinic visits would mean to deviate from the treatment guidelines and add substantially to their own workload. These participants felt that it was important not to turn iCBT into a “blended” treatment, where it is supplemented by components of conventional, face-to-face CBT.

#### 3.4.3 Written communication entails new therapeutic challenges

In line with the therapists' expectations pre-implementation, using iCBT led to a marked shift in the therapeutic relationship compared to conventional CBT. Several participants expressed that it was more difficult to establish an alliance and working relationship at a distance, which they thought may be negative for some patients. According to these participants, the relationship itself can be a mechanism of therapeutic change that is relevant also to digital treatments. As the sole means of communication, the messaging function was seen by all participants as a very important part of the treatment model. Messaging was perceived to constitute a necessary channel for explaining, encouraging, investigating implementation of behavioral strategies and tailoring the treatment to the individual needs of the patient. Through the messaging function, therapists tried to form and sustain working alliances, hoping this would increase chances of the patient completing the treatment.

Communicating with patients in writing also brought challenges. Several participants expressed concern that text messages conveyed less information than a conversation in the consulting room, partly since body language, tone of voice, and other relational signals were absent. The participants outlined several strategies for managing these potential problems, such as expressing themselves more cautiously, using clear and concise language to reduce the risk of misinterpretation. Some mentioned the possibility to consult colleagues for feedback on their written communication.

Contrary to these concerns, some participants reported that the communication and written material produced by certain patients was richer than in standard, face-to-face CBT. They described a category of patients for whom self-disclosure in the consulting room was difficult, but who were more open to share their secret and/or shameful feelings and thoughts in writing. Most participants, however, agreed that they were lacking feedback from the patients on their thoughts, feelings, and experiences of using the program. Many patients were reported to communicate very rarely – if at all. Moreover, with this type of iCBT, communication is asynchronous, meaning that a message might not be read until several days after it was sent, and several more days might pass before a reply is sent. They agreed that in the consulting room, it is common to identify and rectify communication problems, such as patients not quite understanding a metaphor, failing to grasp a concept, or losing motivation. In iCBT, on the other hand, the lack of non-verbal cues and the long time gaps in communication can mean that such problems go unnoticed, thus preventing the therapists from providing adequate support.

The feedback from the patient is missing. You don't see if she is angry or sad – [you don't see] emotional reactions, anxiety levels, symptoms, and so on. And when you don't know where the patient is at, you can't adjust your support to her needs. – Participant 6, post-interview.

## 4 Discussion

The present study specifically aimed to explore the transition from working with face-to-face treatment to using iCBT for youths suffering from anxiety, treated in a primary care context. Therapists' attitudes were examined on two occasions: before starting to use iCBT and at a subsequent follow-up after gaining treatment experience. This is the first published study exploring the transition of working with face-to-face treatment to using iCBT for youths suffering from anxiety treated in primary care. Our findings contribute to new knowledge and highlights several aspects, when implementing iCBT for youths, in the achieved results of the analysis, summed up in four key points, in line with the research questions of the study:

1. What are the attitudes and experiences to iCBT among practicing therapists before and after implementation of iCBT for youths in primary care? Therapists generally had a positive attitude to iCBT for youths and saw it as a valuable alternative to face-to-face treatments.
2. *What are the challenges to consider while implementing iCBT for youths in primary care?* Identified challenges were related to patient selection, and to motivating patients and maintaining a therapeutic relationship through mainly written communication.
3. *What are the experienced changes in the transition from face-to-face therapy to iCBT for youths in primary care?* The participants appreciated the increase in variety that iCBT brought to their schedules, and also experienced iCBT as a relief from common challenges of therapeutic work, such as emotional stress and high cognitive demands.
4. *What are the main factors to consider when implementing iCBT for youths in primary health care?* The participating therapists' positive experiences support the introduction of iCBT for youths in routine primary care. Health care organizations should prioritize iCBT as equal to face-to-face. Parental support is valuable, supplementary treatment components directed at parents seem to be important for compliance and good treatment results.

Based on the present study's results the implementing organization needs a clear understanding of why and how iCBT is going to be implemented in their clinical setting. Furthermore, adequate training in iCBT is paramount and patient assessment and motivation are essential for therapists' experiences confidence when treating adolescents with iCBT.

Before implementation, most participants expressed a strong confidence in iCBT. After gaining treatment experience, they considered iCBT as an effective, accessible, non-stigmatizing, and quality-assured treatment for some, but not all, patients. The participants described iCBT as a potentially resource-efficient method that can enable therapists to provide care to a larger number of patients and they also believed that iCBT can supplement primary care treatment. Before starting to use iCBT, the therapists thought that practitioners would need to learn new skills and also work with a sufficient volume of patients to attain time efficiency. In the post-implementation interviews, this was confirmed and clearly underlined.

All participants said that a prerequisite for successful implementation of iCBT is that the health care organization and management should prioritize iCBT on the same level as conventional treatment. Some participants expressed concern that iCBT work may otherwise be neglected, postponed, or expected to be carried out in gaps between other tasks. All in all, the arguments put forward by the participants for successful implementation of iCBT for youths in primary care are broadly in line with the success factors identified by previous research (Titov et al., 2018), such as that management and organization prioritize implementation.

Although there still is a striking lack of verified predictors of outcome in iCBT (Webb et al., 2017), the participants strongly expressed that comorbidity can be a complicating factor, and that patients with more severe anxiety disorders may need more resource-intensive interventions, such as face-to-face CBT or referral to specialist psychiatric care. This view is in line with research by Haug et al. (2015), which shows that patients with complicated psychopathology and comorbidity typically do not benefit from low-intensity treatment models.

According to the participants in the current study, parents can fill a structuring and motivating function that some patients need, perhaps, especially younger adolescents. To make parental support a reality, however, therapists often needed to motivate both patients and parents. Regarding this purpose, the participants expressed an unmet need for guidelines, materials, and instructions aimed at facilitating parental involvement. There are indications that parental involvement may help increase treatment adherence in iCBT for adolescents (Lenhard et al., 2016).

The participants expressed the concern that adopting iCBT meant having to adopt a new therapist role. The existing research on alliance in iCBT is limited, but indicates that an alliance between therapist and patient correlates positively with treatment outcomes (Pihlaja et al., 2018). This finding is supported by the present study, which clearly points to the importance of continuous communication between therapists and their patients. The participating therapists reported a strong connection between treatment success and an active therapist stance, emphasizing the importance of clarification, validation, and encouragement, as well as individualized tailoring of the treatment. ICBT as described in this study is only one of several ways to deliver digital CBT. Videoconferencing therapy is another solution that is more similar to traditional formats, where therapist and client meet up at a therapist office. It is possible that other digital formats, or combinations of iCBT with these, can provide solutions to some of the issues addressed by therapists in this study, such as difficulties reading emotions.

Several participants expressed a need for improved means of communication, some suggesting real-time chat as an option. This novel kind of “blended” iCBT has been evaluated in several randomized controlled trials, showing highly promising results (Lindqvist et al., 2020; Topooco et al., 2018; Topooco et al., 2019). It is hypothesized that the use of chatting rather than asynchronous emailing is especially well suited to adolescents, given their existing strong tendency to engage in daily online chatting with friends (Topooco et al., 2018). A recent study showed that chating-sessions and extra learning support might enhance treatment effects of iCBT for adolescents with anxiety (Berg et al., 2020). This current study suggests that therapists working in primary care may also be willing to make the transition to instant messaging.

The small sample of participants (n = 14) is a limitation and ideally a larger number of therapists should have been interviewed. Still, although the sample size is modest, data saturation was reached, and no new information came to the fore in the last three interviews. Internet-delivered CBT was still a fairly new intervention tool for the participants. However, the early experiences when implementing a new tool are important because they probably will affect the outcome of the implementation. It is nonetheless important to note that most participants have used no other iCBT program besides *Anxiety Help for Adolescents.* It would be interesting, in the future, to compare results with therapists using other programs. Also, as we did not use closed questions, we cannot give exact numbers of respondents agreeing with an issue.

Research in implementation of iCBT for youths is still in its infancy and strategies for optimizing implementation need to be further developed.

## 5 Conclusion

The present study in a primary care context aimed to explore the transition from working with face-to-face CBT for adolescent anxiety, to treating youths suffering from anxiety with iCBT.

The transition can be challenging, and consequently, therapists need proper training and the organization has to have a clear structure and motivation regarding why, and how, iCBT is implemented. These provisos aside, the therapists appreciated the increased variety iCBT brought to their schedules, and also experienced iCBT as a relief from common effects of therapeutic work, such as emotional stress and high cognitive demands. In sum, the study shows that the interviewed participants generally had positive attitudes to iCBT before starting to use iCBT for youths; at a follow-up after gaining treatment experience, they saw iCBT as a valuable alternative to face-to-face treatments.

## Abbreviations

ACT: Acceptance and Commitment Therapy
CBT: cognitive behavioral therapy
ERP: exposure with response prevention
FoU: *Forskning och Utveckling* (Research and Development), the Swedish Research Register
iCBT: Internet-based cognitive behavioral therapy
IT: information technology
STP: Support and Treatment Platform

## Funding

This study was sponsored by R&D Primary Health Care, 10.13039/100007212Region Västra Götaland. R&D Primary Health Care had no role in the design of the study, the data collection or analysis of data, the writing of the report, or the decision to submit the article for publication.

## Declaration of competing interest

The authors declare the following financial interests/personal relationships which may be considered as potential competing interests: Kristofer Vernmark is employed at Psykologpartners AB which is the company that have developed the treatment program used in this study. The other authors have no conflict of interest.
